# Green-Synthesized TiO_2_ Nanoparticles Improve Mechanical Performance of Glass Ionomer Cements

**DOI:** 10.3390/polym18020295

**Published:** 2026-01-22

**Authors:** Nevra Karamüftüoğlu, Süha Kuşçu, İpek Kuşçu, Nesrin Korkmaz

**Affiliations:** 1 Department of Pediatric Dentistry, Gülhane Faculty of Dentistry, Health Sciences University, Etlik Street, Emrah District, Keçiören, Ankara 06018, Turkey; 2 Department of Prosthodontics, Faculty of Dentistry, Yozgat Bozok University, Ankara Street No. 20, Şeyh Osman District, Yozgat 66200, Turkey; suha.kuscu@yobu.edu.tr (S.K.); a.ipek.kuscu@yobu.edu.tr (İ.K.); 3 Department of Basic Sciences and Health, Hemp Institute, Yozgat Bozok University, Çapanoğlu, Azizli, Yozgat 66200, Turkey; nesrinokumus@gmail.com

**Keywords:** glass ionomer cement, TiO_2_ nanoparticles, green synthesis, hemp extract (*Cannabis sativa*), mechanical properties, flexural strength, microhardness

## Abstract

Glass ionomer cements (GICs) are widely used in restorative and luting dentistry due to their fluoride release and chemical adhesion to dental tissues; however, their limited mechanical strength necessitates reinforcement strategies. The objective of this study was to investigate the effects of hemp-derived, green-synthesized titanium dioxide (TiO_2_) nanoparticles on the surface and mechanical properties of two commercially available GICs with different clinical indications. TiO_2_ nanoparticles were synthesized using *Cannabis sativa* leaf extract via a biogenic reduction method and characterized by X-ray diffraction (XRD), scanning electron microscopy (SEM), and energy-dispersive X-ray spectroscopy (EDX), confirming anatase-phase crystallinity, spherical morphology, and nanoscale particle size (28–49 nm). The nanoparticles were incorporated into Ketac™ Molar Easymix (restorative) and Ketac™ Cem Radiopaque (luting) GICs at 1%, 3%, and 5% (*w*/*w*), with nanoparticle-free formulations serving as controls (n = 10). Surface roughness, Vickers microhardness, and flexural strength were evaluated. Surface roughness increased in a concentration-dependent manner in both materials, with the highest values observed at 5% TiO_2_ incorporation. In Ketac™ Molar Easymix, 1% and 3% TiO_2_ significantly enhanced flexural strength and microhardness, whereas 5% resulted in reduced performance, consistent with SEM-observed nanoparticle agglomeration. In contrast, Ketac™ Cem Radiopaque exhibited no significant changes in flexural strength, although maximum microhardness values were recorded at 1% TiO_2_ concentration. These findings demonstrate that low concentrations of hemp-derived TiO_2_ nanoparticles can effectively reinforce restorative GICs and highlight the potential of green nanotechnology as a sustainable approach for improving dental biomaterials.

## 1. Introduction

Glass ionomer cements (GICs), first introduced by Wilson and Kent in 1969, are bioactive dental materials formed through an acid–base reaction between fluoroaluminosilicate glass powder and polycarboxylic acids [1]. Due to their chemical adhesion to dental hard tissues, fluoride-releasing ability, and favorable biocompatibility, GICs have been extensively used in restorative dentistry, luting procedures, and preventive applications [2]. Their clinical versatility has made them particularly valuable in pediatric and minimally invasive dentistry [3]. Despite these advantages, conventional GICs present notable mechanical shortcomings, including low flexural strength, brittleness, and susceptibility to wear and moisture sensitivity, which limit their long-term clinical durability [3,4]. These limitations have been consistently highlighted in the literature and remain a primary concern restricting the broader use of GICs in stress-bearing areas [4,5]. To improve the mechanical performance of GICs, various reinforcement strategies have been explored. Among these approaches, modifications of the glass composition and incorporation of reinforcing phases have been proposed to enhance strength and durability while preserving fluoride release and adhesion [5]. Recent research trends increasingly focus on sustainable and bio-based reinforcement strategies for dental materials [6].

Hemp (*Cannabis sativa*) has attracted growing attention as a sustainable reinforcing material due to its high cellulose content, biodegradability, and low environmental impact [7]. Hemp-derived fibers and additives have been successfully incorporated into composite systems, demonstrating improvements in mechanical performance and environmental sustainability [7,8]. In dental materials research, hemp-based reinforcements have recently been reported to enhance the mechanical properties of glass ionomer cements, highlighting their potential as eco-friendly alternatives to synthetic fillers [6].

In parallel, the incorporation of inorganic fillers, particularly at the nanoscale, has emerged as an effective strategy for reinforcing GICs. Titanium dioxide (TiO_2_) nanoparticles are widely investigated due to their chemical stability, biocompatibility, and favorable interaction with cement matrices [9]. Previous studies have demonstrated that TiO_2_ nanoparticle incorporation can improve the physical, chemical, and biological properties of GICs, although these effects are highly dependent on nanoparticle concentration and dispersion [9]. The reinforcing efficiency of fillers in GICs is strongly influenced by particle size, surface characteristics, and interaction with the glass matrix. Variations in aluminosilicate glass composition and filler characteristics have been shown to significantly affect the mechanical behavior of GICs, emphasizing the importance of controlled filler incorporation [10]. While nanoparticle reinforcement offers clear advantages, the synthesis method of nanoparticles has become an important consideration. Conventional nanoparticle synthesis routes often rely on chemical processes associated with environmental and health concerns [11]. As an alternative, green synthesis methods using biological systems have gained increasing attention due to their reduced toxicity, lower energy requirements, and environmental compatibility [12]. Biomolecule-assisted green synthesis utilizes natural reducing and stabilizing agents to produce nanoparticles suitable for biomedical applications. Such approaches enable the formation of stable metal and metal oxide nanoparticles while minimizing hazardous by-products, making them particularly attractive for dental biomaterials research [13].

To date, however, no study has evaluated the use of hemp-mediated, green-synthesized TiO_2_ nanoparticles as reinforcing agents in glass ionomer cements. Furthermore, comparative assessments of their effects on surface roughness, microhardness, and flexural strength in both restorative and luting GICs remain absent from the current literature. Addressing this gap, the present study investigates the effects of incorporating green-synthesized TiO_2_ nanoparticles into two types of GICs (restorative and luting), focusing on their impact on surface roughness, microhardness, and flexural strength. This study aims to expand current knowledge on sustainable reinforcement strategies and explore the potential of plant-derived nanomaterials in dental biomaterial engineering.

The novelty of the present study lies in the use of hemp (*Cannabis sativa*) extract as a biological mediator for the green synthesis of TiO_2_ nanoparticles and their subsequent incorporation into glass ionomer cements. Although hemp-based reinforcements and TiO_2_ nanoparticle-modified GICs have been investigated independently in previous studies, their integration through a plant-mediated nanoparticle synthesis approach in both restorative and luting glass ionomer cements has not been previously reported. TiO_2_ nanoparticles were selected due to their established biocompatibility, chemical stability, and documented reinforcing effects in glass ionomer cement matrices, while hemp extract was chosen as a green synthesis medium because of its sustainability, low environmental impact, and ability to facilitate biomolecule-assisted nanoparticle formation. Accordingly, the primary objective of this study was to evaluate the effects of hemp-mediated, green-synthesized TiO_2_ nanoparticles incorporated at concentrations of 1%, 3%, and 5% (*w*/*w*) on the surface roughness, microhardness, and flexural strength of two commercially available glass ionomer cements with different clinical indications (restorative and luting), while the secondary objective was to investigate the relationship between nanoparticle concentration and mechanical performance in order to identify an optimal reinforcement level within a sustainable dental biomaterial framework. The null hypothesis (H_0_) of this study was that the incorporation of hemp-derived, green-synthesized TiO_2_ nanoparticles at different concentrations (1%, 3%, and 5% *w*/*w*) would not result in statistically significant differences in surface roughness, microhardness, or flexural strength of restorative and luting glass ionomer cements compared with nanoparticle-free control groups.

## 2. Materials and Methods

### 2.1. Materials

Two commercially available conventional glass ionomer cements (GICs) were used in this study: a restorative GIC (Ketac™ Molar Easymix) and a luting GIC (Ketac™ Cem Radiopaque) (3M ESPE Dental Products, St. Paul, MN, USA). Ketac™ Molar Easymix is a high-viscosity conventional GIC indicated for restorative applications, while Ketac™ Cem Radiopaque is a conventional glass ionomer luting cement. Both materials comply with ISO 9917-1:2025 standards for water-based dental cements [14]. The powder-to-liquid ratios were prepared strictly according to the manufacturer’s (3M ESPE Dental Products, St. Paul, MN, USA) instructions. Batch and lot numbers were recorded at the time of specimen preparation.

Titanium dioxide (TiO_2_) nanoparticles were synthesized in-house using a green synthesis approach. Titanium tetrachloride (TiCl_4_, ≥99% purity) used as the titanium precursor was obtained from a commercial chemical supplier (Merck, Darmstadt, Germany). Ammonium hydroxide solution (NH_4_OH, 25% *w*/*w*) used for pH adjustment and precipitation was also supplied by Merck (Darmstadt, Germany). Distilled water was used throughout all synthesis and washing procedures.

Hemp (*Cannabis sativa*) leaves were obtained from the Hemp Research Institute of Yozgat Bozok University (Yozgat, Türkiye). The plant material was taxonomically identified and consisted exclusively of leaf tissue. The leaves were washed, shade-dried under controlled laboratory conditions, and stored in airtight containers prior to extract preparation.

All chemical reagents used in the study were of analytical grade and used without further purification. All materials were handled and stored according to the manufacturer’s (3M ESPE Dental Products, St. Paul, MN, USA) recommendations.

### 2.2. Methods

#### 2.2.1. Preparation of Hemp Extract

Hemp leaves obtained from the Hemp Research Institute of Yozgat Bozok University were washed with tap water followed by distilled water and then dried in a shaded, ventilated environment to preserve phytochemical stability. The dried material was ground using a sterile mortar. A total amount of 10 g of powdered biomass was mixed with 100 mL of distilled water and heated at 60 °C for 2 h under magnetic stirring. After cooling, the mixture was centrifuged at 3500 rpm for 15 min, and the resulting supernatant was stored at 4 °C for nanoparticle synthesis.

#### 2.2.2. Green Synthesis of TiO_2_ Nanoparticles

Biogenic TiO_2_ nanoparticles were synthesized by mixing 80 mL of hemp extract with 80 mL of 0.5 M TiCl_4_ solution (1:1, *v*/*v*) under continuous stirring at room temperature for 4 h. Ammonia solution (15 mL) was added dropwise to adjust the pH and facilitate the formation of a white precipitate. The precipitate was collected by centrifugation (3500 rpm), washed repeatedly with distilled water, and air-dried. Final calcination was performed at 450 °C for 3 h to obtain crystalline TiO_2_ nanoparticles. Particle morphology and structure were characterized using scanning electron microscopy (SEM), energy dispersive X-ray spectroscopy (EDX), and X-ray diffraction (XRD).

#### 2.2.3. Preparation of Experimental Groups

Two commercially available GICs—Ketac™ Cem Radiopaque (luting) and Ketac™ Molar Easymix (restorative) (3M ESPE Dental Products, St. Paul, MN, USA) were used. TiO_2_ nanoparticles were incorporated at concentrations of 1%, 3%, and 5% (*w*/*w*) into the powder phase of each GIC. A nanoparticle-free formulation served as the control, resulting in eight groups (n = 10 per group). All materials were mixed according to the manufacturer’s powder-to-liquid instructions. The cement mixtures were placed into stainless-steel molds (10 mm diameter × 2 mm thickness), covered with acetate strips and glass slides to ensure flat surfaces, and allowed to set. Specimens were ultrasonically cleaned in distilled water and air-dried prior to testing.

#### 2.2.4. Surface Roughness Analysis

Surface roughness (Ra) was measured using a Taylor Hobson Surtronic 25 profilometer (Taylor Hobson Ltd., Leicester, UK) (cut-off: 0.25 mm; traverse speed: 0.5 mm/s). Three to five readings were obtained from the central region of each specimen, and mean Ra values were recorded. Instrument calibration was performed using a 0.2 µm standard reference block.

#### 2.2.5. Flexural Strength Testing

Flexural strength was evaluated following ASTM E399-90 specimen dimensions (25 × 2.5 × 5 mm) with a central notch width of 0.5 mm. After 24 h of storage in distilled water at 37 °C, samples were tested using a Shimadzu AGS-X universal (Shimadzu Corporation, Kyoto, Japan) testing machine at a crosshead speed of 0.5 mm/min. Flexural strength (MPa) was calculated using the maximum load (N) recorded at fracture. Glass ionomer cements exhibited a predominantly brittle fracture behavior during flexural testing. Therefore, flexural strength values were calculated based on the maximum stress recorded at fracture, and continuous stress–strain curves were not generated due to the absence of measurable plastic deformation.

#### 2.2.6. Microhardness Measurement

Vickers microhardness was measured according to ASTM E384-17 using a Shimadzu Dynamic Ultra Microhardness Tester (Shimadzu Corporation, Kyoto, Japan). A load of 200 g was applied through a 40× objective lens. Five indentations were placed on each specimen, and the mean hardness value was calculated. In a subset of samples, a 500 g load applied for 10 s was used to confirm load-dependent behavior based on prior methodological reports. Microhardness values were determined based on the measured indentation diagonals; representative indentation images were not recorded during testing.

#### 2.2.7. SEM and EDX Characterization

Surface morphology and elemental distribution were analyzed using a PHILIPS XL-30 SEM (Philips Electron Optics, Eindhoven, The Netherlands) operated at ×100, ×500, and ×3000 magnifications. Samples were polished sequentially with 400-, 1000-, and 1500-grit silicon carbide papers, rinsed, and ultrasonically cleaned. EDX spectra were obtained to verify elemental composition and confirm TiO_2_ incorporation.

#### 2.2.8. Statistical Analysis

All experimental procedures were performed under standardized laboratory conditions. For each experimental group, ten specimens were prepared (n = 10). Surface roughness measurements were repeated three to five times per specimen, microhardness measurements were performed at five different locations on each specimen, and flexural strength testing was conducted once per specimen following standardized testing protocols. All measurements were carried out by the same operator using calibrated instruments to ensure repeatability and to minimize operator-related variability. Results were expressed as mean values with standard deviations. All statistical analyses were conducted using SPSS version 25.0 (IBM Corp., Armonk, NY, USA). Data normality was assessed using the Shapiro–Wilk test, and homogeneity of variances using Levene’s test. One-way ANOVA followed by Duncan’s post hoc test was applied for normally distributed data, whereas the Mann–Whitney U test was used for non-parametric comparisons. Statistical significance was set at *p* < 0.05.

Sample size selection (n = 10 per group) was based on previously published in vitro studies evaluating the mechanical and surface properties of glass ionomer cements reinforced with nanoparticles. Considering the exploratory and comparative nature of the present study, this sample size was deemed sufficient to detect meaningful differences among groups. A post hoc power consideration indicated that the study had adequate statistical power (≥80%) to detect significant differences in the primary outcome variables.

## 3. Results

### 3.1. XRD Analysis of TiO_2_ Nanoparticles

**Figure 1:**
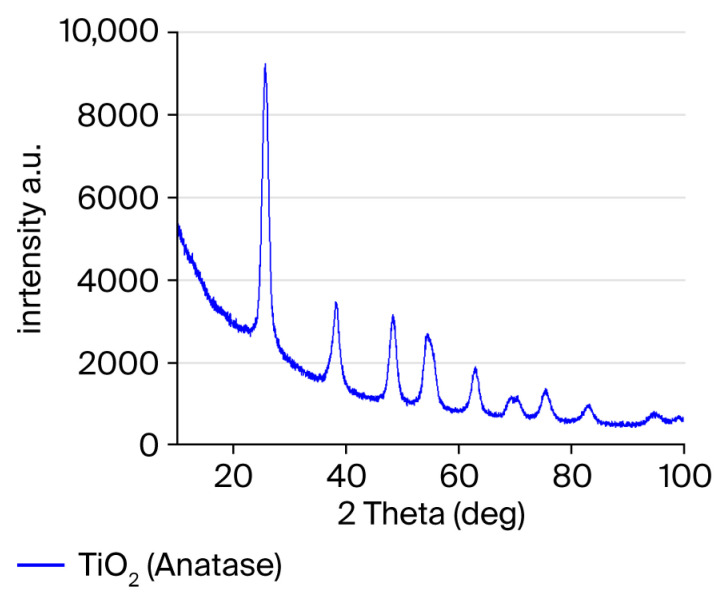
XRD pattern of green-synthesized TiO_2_ nanoparticles.

The crystalline structure of the biogenically synthesized TiO_2_ nanoparticles is presented in Figure 1. Distinct diffraction peaks were detected at 2θ values of 25.62°, 38.11°, 48.33°, 54.51°, 63.09°, 70.17°, and 75.45°, matching the (101), (103), (200), (211), (204), (220), and (215) lattice planes, respectively. The dominant peak at 25.62° confirmed a highly crystalline anatase phase, consistent with reference pattern 98-009-2363.

The average crystallite size was calculated as 44 nm using the Debye–Scherrer equation. No secondary phases or impurities were detected. Although transmission electron microscopy (TEM) analysis could provide direct validation of crystallite size at the individual particle level, it was not performed in the present study. Instead, SEM analysis was used as a complementary technique to XRD, and the particle size range observed by SEM showed close agreement with the crystallite size estimated by the Debye-Scherrer equation, supporting the reliability of the XRD-based calculation. These findings align with previous reports describing green-synthesized TiO_2_ nanoparticles within a similar size range [15,16]. Although Rietveld refinement analysis could provide further quantitative confirmation of phase composition and crystallographic parameters, it was not performed in the present study, as the primary purpose of the XRD analysis was qualitative phase identification. The clear correspondence of all diffraction peaks with the reference anatase TiO_2_ pattern and the absence of secondary phases were considered sufficient to confirm phase purity within the scope of this study.

### 3.2. SEM Analysis of TiO_2_ Nanoparticles

**Figure 2:**
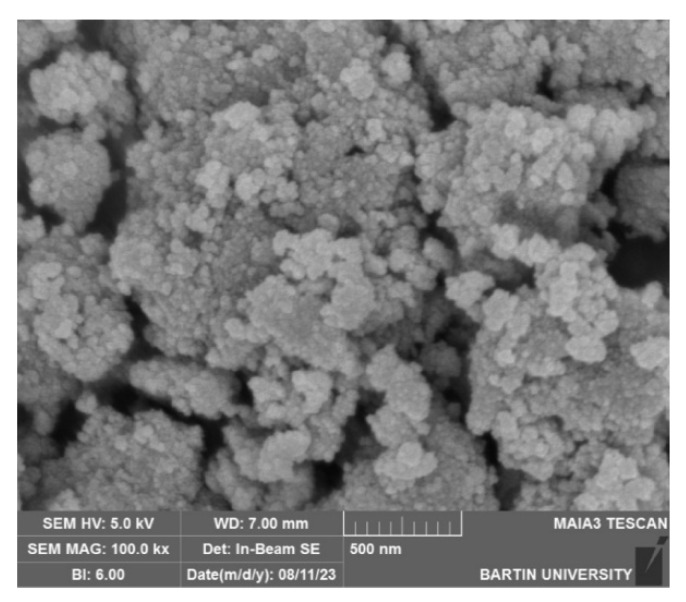
SEM image of green-synthesized TiO_2_ nanoparticles.

SEM micrographs obtained using a Quanta 450 FEG microscope (Figure 2) revealed predominantly spherical nanoparticles with evident agglomeration. Particle dimensions ranged from 28 to 49 nm, showing close agreement with the crystallite size derived from XRD. This consistency between SEM-observed particle size and XRD-derived crystallite size further supports the validity of the Debye-Scherrer estimation in the absence of TEM analysis. The agglomeration observed is a commonly reported phenomenon in plant-mediated TiO_2_ nanoparticle synthesis [15,16,17].

### 3.3. EDX Analysis of TiO_2_ Nanoparticles

**Figure 3:**
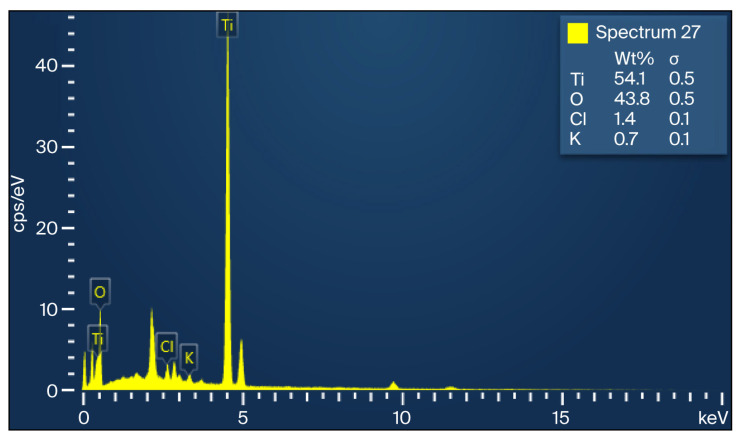
EDX spectrum of green-synthesized TiO_2_ nanoparticles.

EDX spectra (Figure 3) demonstrated that the nanoparticles consisted mainly of titanium (54.1%) and oxygen (43.8%), confirming the formation of TiO_2_. Minor traces of chlorine (1.4%) and potassium (0.7%) were detected, attributable to the TiCl_4_ precursor and phytochemical residues from the plant extract, respectively. Similar elemental profiles have been documented in related studies [15,18].

### 3.4. Surface Roughness

Surface roughness (Ra) values for all groups are summarized in Table 1. In both Ketac™ Cem Radiopaque and Ketac™ Molar Easymix, the incorporation of TiO_2_ nanoparticles resulted in a concentration-dependent increase in surface roughness. One-way ANOVA revealed that these differences were statistically significant among nanoparticle concentrations (*p* < 0.05). Post hoc comparisons demonstrated that the highest Ra values were associated with the 5% TiO_2_ nanoparticle groups in both materials.

- In Ketac™ Cem Radiopaque, the highest Ra values were recorded at 5% NP concentration.
- In Ketac™ Molar Easymix, significant increases were observed at 3% and 5% concentrations.

Overall, nanoparticle addition progressively increased surface irregularities in both materials.

### 3.5. Flexural Strength

*Ketac™ Molar Easymix:*

Flexural strength values are shown in Table 2. The incorporation of TiO_2_ nanoparticles significantly improved flexural strength compared with the control (0% NP concentration). The greatest increases were observed at 1% and 3% concentrations. No significant differences occurred between nanoparticle-containing groups, indicating a plateau effect beyond 1% NP addition.

**Figure 4:**
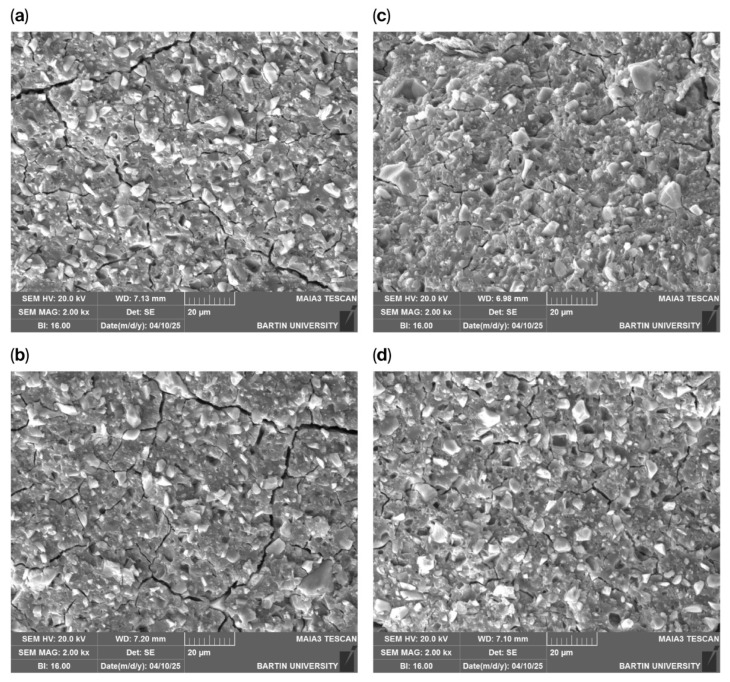
(**a**) Fracture surface SEM image (Ketac™ Molar Easymix, Control group); (**b**) fracture surface SEM image (Ketac™ Molar Easymix, %1 NP); (**c**) fracture surface SEM image (Ketac™ Molar Easymix, %3 NP); (**d**) fracture surface SEM image (Ketac™ Molar Easymix, %5 NP).

SEM fracture surface images (Figure 4) revealed denser microstructures and more pronounced internal features in nanoparticle-reinforced specimens.

*Ketac™ Cem Radiopaque:*

No statistically significant differences in flexural strength were detected between groups (Table 3).

**Figure 5:**
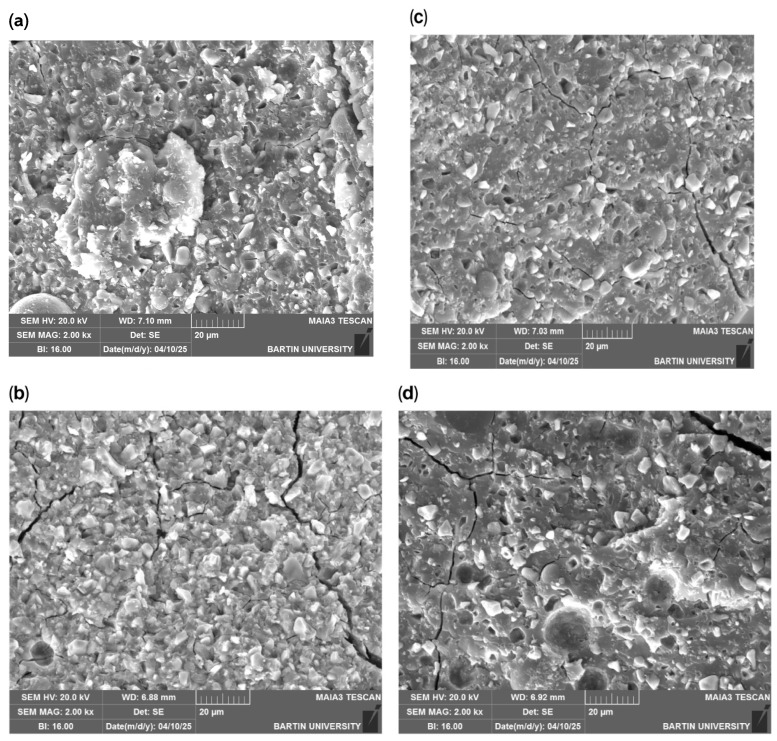
(**a**) Fracture surface SEM image (Ketac™ Cem Radiopaque, Control group); (**b**) fracture surface SEM image (Ketac™ Cem Radiopaque, %1 NP); (**c**) fracture surface SEM image (Ketac™ Cem Radiopaque, %3 NP); (**d**) fracture surface SEM image (Ketac™ Cem Radiopaque, %5 NP).

SEM images (Figure 5) indicated relatively smooth fracture surfaces in control specimens, while higher TiO_2_ concentrations produced more irregular topographies.

#### Vickers Microhardness

Microhardness values for all materials are presented in Table 4.

- Ketac™ Molar Easymix: Significant increases were observed at 1% and 3% TiO_2_, while slight reductions occurred at a 5% NP concentration.
- Ketac™ Cem Radiopaque: The highest hardness was recorded at 1% NP concentration, with decreases at higher concentrations (3% and 5%).

Across both materials, low nanoparticle concentrations improved microhardness, whereas higher loadings likely resulted in agglomeration, reducing reinforcement efficiency.

## 4. Discussion

This study provides a comprehensive evaluation of the effects of hemp-derived, green-synthesized titanium dioxide nanoparticles on the physical and mechanical properties of two different glass ionomer cement systems. The findings demonstrate that biogenically synthesized TiO_2_ nanoparticles can modify GIC performance in a concentration-dependent and material-specific manner, offering a sustainable reinforcement strategy consistent with the principles of eco-friendly dental material development. The increase in surface roughness observed in both Ketac™ Molar Easymix and Ketac™ Cem Radiopaque, particularly at a 5% nanoparticle concentration, is likely attributable to nanoparticle agglomeration and subsequent microstructural irregularities. The SEM micrographs clearly demonstrated increased nanoparticle agglomeration at higher TiO_2_ concentrations, which provides a microstructural explanation for the observed deterioration in certain mechanical properties. Agglomerated nanoparticles tend to form non-uniform clusters within the cement matrix, reducing effective particle dispersion and limiting the available surface area for interaction with the polycarboxylate matrix. As a result, these clusters may act as stress concentration sites, facilitating crack initiation and propagation under mechanical loading. This phenomenon explains the plateau or reduction in flexural strength and microhardness observed at a 5% nanoparticle concentration, despite the reinforcing potential of TiO_2_ at lower loadings. Therefore, the mechanical performance of TiO_2_-reinforced glass ionomer cements is not solely dependent on nanoparticle presence but is critically governed by dispersion quality and agglomeration behavior within the cement matrix. Comparable increases in roughness have been reported for TiO_2_-modified GICs in previous investigations, which similarly attributed these changes to particle clustering and disrupted matrix uniformity [16,19]. Since rougher surfaces are known to promote plaque accumulation and bacterial adhesion [20], optimizing the nanoparticle concentration is essential to ensure a favorable balance between reinforcement and clinical surface quality. In addition to its role in bacterial adhesion, surface roughness is closely associated with the biological response and adhesion behavior of glass ionomer cements. Moderate increases in surface roughness may enhance micromechanical interlocking and contribute positively to adhesion to dental hard tissues, particularly when combined with the intrinsic chemical bonding mechanism of GICs. However, excessive surface roughness may adversely affect biocompatibility by facilitating biofilm maturation and inflammatory responses at the tooth-material interface. Therefore, the concentration-dependent increase in surface roughness observed in the present study suggests that while low TiO_2_ nanoparticle loadings may support mechanical performance without compromising biological compatibility, higher concentrations could pose potential risks for long-term clinical adhesion and tissue response. In addition to its role in bacterial adhesion, surface roughness is closely associated with the biological response and adhesion behavior of glass ionomer cements. Moderate increases in surface roughness may enhance micromechanical interlocking and contribute positively to adhesion to dental hard tissues, particularly when combined with the intrinsic chemical bonding mechanism of GICs. However, excessive surface roughness may adversely affect biocompatibility by facilitating biofilm maturation and inflammatory responses at the tooth-material interface. Therefore, the concentration-dependent increase in surface roughness observed in the present study suggests that while low TiO_2_ nanoparticle loadings may support mechanical performance without compromising biological compatibility, higher concentrations could pose potential risks for long-term clinical adhesion and tissue response.

Although TiO_2_ nanoparticles and green synthesis approaches have been independently investigated in previous studies, the present work provides an incremental yet meaningful contribution by integrating a hemp (*Cannabis sativa*)–mediated synthesis route with a comparative evaluation of both restorative and luting glass ionomer cements within the same experimental framework. This dual-material approach enables a clearer understanding of how nanoparticle reinforcement behaves across different clinical indications, thereby extending existing protocols toward a sustainability-oriented and application-specific perspective.

Enhancements in flexural strength were evident only in the restorative GIC (Ketac™ Molar Easymix), with significant improvements at 1% and 3% TiO_2_ loading. These findings reflect the established principle that low nanoparticle concentrations can strengthen brittle materials by promoting crack deflection and improving stress distribution, whereas higher concentrations result in diminishing returns due to agglomeration and matrix discontinuity. The plateau observed beyond 1% is consistent with the outcomes reported by Ramić et al. [21]. In contrast, Ketac™ Cem Radiopaque did not exhibit any statistically significant change in flexural strength across nanoparticle concentrations. The lack of flexural strength enhancement in Ketac™ Cem Radiopaque may be attributed to material-specific chemical and structural characteristics. As a luting glass ionomer cement, Ketac™ Cem Radiopaque contains a lower powder-to-liquid ratio and a different aluminosilicate glass composition compared with restorative GICs, which may restrict effective interaction between TiO_2_ nanoparticles and the polycarboxylate matrix. In addition, the rapid acid–base reaction and thinner cement matrix typical of luting cements may limit nanoparticle participation in stress transfer and crack-bridging mechanisms, thereby reducing their reinforcing efficiency. This material-specific response supports earlier reports suggesting that the reinforcement potential of TiO_2_ nanoparticles depends on the intrinsic composition, filler ratio, and acid–base reaction dynamics of each GIC formulation [22]. While flexural strength provides valuable information on the load-bearing capacity of glass ionomer cements, it does not fully describe crack initiation and propagation behavior. Fracture toughness is particularly relevant for brittle materials such as GICs, as it reflects resistance to crack growth under stress. Although fracture toughness was not evaluated in the present study, the observed improvements in flexural strength at 1% and 3% TiO_2_ concentrations suggest a potential enhancement in crack resistance, possibly due to improved stress distribution and crack deflection mechanisms at low nanoparticle loadings. Future studies incorporating fracture toughness measurements would be beneficial to further elucidate the reinforcing mechanisms of green-synthesized TiO_2_ nanoparticles in glass ionomer cement systems. These findings suggest that the reinforcing potential of TiO_2_ nanoparticles is highly dependent on cement formulation and is more pronounced in restorative glass ionomer systems than in luting formulations.

Microhardness values increased significantly at 1% and 3% TiO_2_ concentration for both materials, with slight reductions at 5%. The reduction in microhardness observed at a 5% TiO_2_ concentration can be attributed to nanoparticle agglomeration, which disrupts matrix continuity and limits effective stress transfer at the indentation site. At lower concentrations, well-dispersed nanoparticles may act as reinforcement centers, enhancing resistance to localized deformation. In contrast, excessive nanoparticle loading promotes cluster formation, resulting in voids and heterogeneities that compromise surface integrity and reduce hardness values. This decrease aligns with the expected negative influence of particle agglomeration, which interferes with the polymeric network and reduces localized hardness. These outcomes are consistent with previously published data showing beneficial effects of low-concentration nanoparticle incorporation and performance decline at higher levels [19,23]. Increased microhardness is particularly relevant for restorative applications, as it indicates improved wear resistance and surface durability.

The characterization results further supported the mechanical findings. XRD confirmed the anatase crystalline phase of the TiO_2_ nanoparticles, while SEM micrographs demonstrated nanoscale spherical morphology with partial agglomeration at higher concentrations. The agreement between crystallite size and SEM-derived particle size range indicates stable synthesis conditions. Elemental verification through EDX confirmed the expected Ti and O composition with minor plant-derived residues. These structural and compositional features suggest that the nanoparticles successfully integrated into the GIC matrix and likely interacted with the polycarboxylate chains, influencing mechanical behavior in a concentration-dependent fashion. Beyond initial crystallinity and morphology, the stability of TiO_2_ nanoparticles within the glass ionomer cement matrix over time is a critical factor for sustained material performance. TiO_2_ nanoparticles are known for their chemical inertness, high thermodynamic stability, and resistance to dissolution under oral environmental conditions. When embedded within the crosslinked polycarboxylate matrix of glass ionomer cements, these nanoparticles are expected to remain structurally stable and retain their reinforcing potential, particularly due to the strong ionic interactions between the cement matrix and the aluminosilicate glass network. Although long-term aging or degradation analyses were not performed in the present study, previously reported investigations suggest that TiO_2_ nanoparticles maintain structural integrity within GIC systems over time. Nevertheless, future studies incorporating artificial aging, thermal cycling, and long-term storage conditions are warranted to fully elucidate the time-dependent stability of green-synthesized TiO_2_ nanoparticles in glass ionomer cements.

Although antibacterial activity was not directly investigated in the present study, previous studies have reported antimicrobial effects of TiO_2_ nanoparticles in various dental material systems [23]. Therefore, any reference to potential antibacterial behavior in this study should be interpreted solely as a literature-based consideration rather than a demonstrated outcome. The present findings are limited to the evaluation of mechanical and surface properties, and future studies incorporating standardized antibacterial assays are required to determine whether hemp-derived TiO_2_ nanoparticle–modified glass ionomer cements exhibit clinically relevant antimicrobial activity.

In addition to the mechanical and structural insights, an important contribution of this work lies in its use of plant-mediated nanoparticle synthesis. Green synthesis processes minimize toxic chemicals, reduce energy consumption, and improve environmental safety. Given the growing emphasis on sustainability in biomaterials research, the production of TiO_2_ nanoparticles from hemp provides a model for environmentally responsible innovation in dental materials science. From a sustainability perspective, green-synthesized TiO_2_ nanoparticles offer potential advantages over conventionally synthesized counterparts, including reduced use of hazardous chemicals, lower energy consumption, and improved environmental compatibility. Although the present study did not include a direct comparison between green-synthesized and chemically synthesized TiO_2_ nanoparticles, future studies incorporating comparative mechanical performance, environmental impact assessments, and life-cycle analyses would provide valuable insight into the broader sustainability benefits of bio-mediated nanoparticle synthesis in dental materials.

Overall, the findings demonstrate that hemp-derived TiO_2_ nanoparticles can reinforce glass ionomer cements at optimal concentrations, particularly in restorative GICs. However, the reinforcement effect is dependent on nanoparticle distribution, concentration, and intrinsic material characteristics. Future investigations should assess long-term biocompatibility, fluoride release, antibacterial activity, wear behavior, and clinical durability of these materials. Additionally, comparative environmental impact analyses between green-synthesized and conventionally synthesized nanoparticles would further advance the field of sustainable dental biomaterials.

## 5. Conclusions

- Hemp-mediated green synthesis enabled the successful production of TiO_2_ nanoparticles and their incorporation into restorative and luting glass ionomer cements.
- Low nanoparticle concentrations (1% and 3%) enhanced flexural strength and microhardness in the restorative GIC, whereas higher loading (5%) resulted in performance reduction, likely due to nanoparticle agglomeration.
- Surface roughness increased in a concentration-dependent manner for both materials, highlighting the importance of optimizing nanoparticle concentration to balance mechanical reinforcement and clinical surface quality.
- No significant improvement in flexural strength was observed for Ketac™ Cem Radiopaque, which may be attributed to material-specific compositional and matrix-related factors.
- The reinforcing efficiency of TiO_2_ nanoparticles was strongly dependent on cement formulation and nanoparticle dispersion within the matrix.
- Although antibacterial activity and advanced mechanical properties were not directly evaluated, future studies incorporating tensile behavior, fracture-related parameters, antibacterial assays, and environmental impact analyses are warranted to further elucidate the multifunctional and sustainable potential of green-synthesized TiO_2_-reinforced glass ionomer cements.

## Figures and Tables

**Table 1:** Surface roughness (Ra) values of GIC groups.

Material	Group	Mean Ra (µm)	Standard Deviation (µm)	Minimum Ra (µm)	Maximum Ra (µm)
Ketac™ Cem Radiopaque	Control (No NP)	0.42	0.29	0.01	0.84
	1% TiO_2_ NP	0.69	0.34	0.22	1.16
	3% TiO_2_ NP	0.74	0.38	0.26	1.24
	5% TiO_2_ NP	0.98	0.40	0.58	1.38
Ketac™ Molar Easymix	Control (no NPs)	0.80	0.45	0.01	1.60
	1% TiO_2_ NP	1.10	0.38	0.56	1.42
	3% TiO_2_ NP	1.20	0.42	0.50	1.74
	5% TiO_2_ NP	1.18	0.44	0.48	1.72

**Table 2:** Flexural strength (MPa) pairwise comparison of Ketac™ Molar Easymix groups.

No.	Group	*p*-Value	Significance	Mean Flexural Strength (MPa)	Std Dev Flexural Strength (MPa)
**1**	0.0 vs. 0.01	0.0281	Significant	1.3324	0.1864
**2**	0.0 vs. 0.03	0.0206	Significant	0.7123	0.1582
**3**	0.0 vs. 0.05	0.0207	Significant	0.6818	0.2224
**4**	0.01 vs. 0.03	0.743	Not Significant	0.6834	0.1279
**5**	0.01 vs. 0.05	0.7209	Not Significant	0.8042	0.1584
**6**	0.03 vs. 0.05	0.673	Not Significant	1.0248	0.1733

**Table 3:** Flexural strength (MPa) pairwise comparison of Ketac™ Cem Radiopaque groups.

No.	Group	*p*-Value	Significance	Mean Flexural Strength (MPa)	Std Dev Flexural Strength (MPa)
**1**	0.0 vs. 0.01	0.152	Not Significant	0.8745	0.1116
**2**	0.0 vs. 0.03	0.9182	Not Significant	1.4507	0.2732
**3**	0.0 vs. 0.05	0.152	Not Significant	1.232	0.2202
**4**	0.01 vs. 0.03	0.0745	Not Significant	1.0987	0.2416
**5**	0.01 vs. 0.05	0.8785	Not Significant	0.656	0.1041
**6**	0.03 vs. 0.05	0.0592	Not Significant	0.656	0.294

**Table 4:** Vickers microhardness values of experimental groups.

Material	Group	Measurement 1	Measurement 2	Measurement 3	Mean ± SD
Easymix	Control (0% NP)	48.6	38.4	42.6	43.2 ± 5.1
	0-2	34.9	34.2	38.2	35.8 ± 2.1
	1-1 (%1 NP)	38.4	37.1	39.7	38.4 ± 1.3
	1-2 (%1 NP)	40.8	46.0	42.9	43.2 ± 2.6
	3-1 (%3 NP)	41.5	39.7	45.8	42.3 ± 3.1
	3-2 (%3 NP)	44.4	44.7	46.4	45.2 ± 1.0
	5-1 (%5 NP)	30.2	38.8	36.4	35.1 ± 4.3
	5-2 (%5 NP)	34.8	35.7	30.5	33.7 ± 2.6
Cem Radiopak	Control (0% NP)	52.1	43.5	48.0	47.9 ± 4.3
	0-2	38.7	50.8	47.4	45.6 ± 6.1
	1-1 (%1 NP)	75.8	87.9	80.8	81.5 ± 6.1
	1-2 (%1 NP)	69.4	73.8	75.8	73.0 ± 3.2
	3-1 (%3 NP)	70.4	41.0	72.3	61.2 ± 16.7
	5-1 (%5 NP)	58.3	64.1	73.0	65.1 ± 7.4
	5-2 (%5 NP)	53.8	64.1	58.0	58.6 ± 5.2

## Data Availability

The original contributions presented in this study are included in the article. Further inquiries can be directed to the corresponding author.
